# Comparative chronic toxicity of three neonicotinoids on New Zealand packaged honey bees

**DOI:** 10.1371/journal.pone.0190517

**Published:** 2018-01-02

**Authors:** Sarah C. Wood, Ivanna V. Kozii, Roman V. Koziy, Tasha Epp, Elemir Simko

**Affiliations:** 1 Department of Veterinary Pathology, Western College of Veterinary Medicine, University of Saskatchewan, Saskatoon, Saskatchewan, Canada; 2 Department of Large Animal Clinical Sciences, Western College of Veterinary Medicine, University of Saskatchewan, Saskatoon, Saskatchewan, Canada; Philipps-Universitat Marburg Fachbereich Biologie, GERMANY

## Abstract

**Background:**

Thiamethoxam, clothianidin, and imidacloprid are the most commonly used neonicotinoid insecticides on the Canadian prairies. There is widespread contamination of nectar and pollen with neonicotinoids, at concentrations which are sublethal for honey bees (*Apis mellifera* Linnaeus).

**Objective:**

We compared the effects of chronic, sublethal exposure to the three most commonly used neonicotinoids on honey bee colonies established from New Zealand packaged bees using colony weight gain, brood area, and population size as measures of colony performance.

**Methods:**

From May 7 to July 29, 2016 (12 weeks), sixty-eight colonies received weekly feedings of sugar syrup and pollen patties containing 0 nM, 20 nM (median environmental dose), or 80 nM (high environmental dose) of one of three neonicotinoids (thiamethoxam, clothianidin, and imidacloprid). Colonies were weighed at three-week intervals. Brood area and population size were determined from digital images of colonies at week 12. Statistical analyses were performed by ANOVA and mixed models.

**Results:**

There was a significant negative effect (-30%, p<0.01) on colony weight gain (honey production) after 9 and 12 weeks of exposure to 80 nM of thiamethoxam, clothianidin, or imidacloprid and on bee cluster size (-21%, p<0.05) after 12 weeks. Analysis of brood area and number of adult bees lacked adequate (>80%) statistical power to detect an effect.

**Conclusions:**

Chronic exposure of honey bees to high environmental doses of neonicotinoids has negative effects on honey production. Brood area appears to be less sensitive to detect sublethal effects of neonicotinoids.

## Introduction

Neonicotinoids are the youngest, and arguably the most safe, effective, and widely used class of neuroactive insecticides worldwide [1, 2]. Thiamethoxam (THI), clothianidin (CLO), and imidacloprid (IMD) are the most commonly used neonicotinoid insecticides on the Canadian prairies where they are applied as a seed treatment to a variety of crops, including greater than 95% of canola [3, 4]. When used as a prophylactic seed treatment, the neonicotinoid is taken up into the growing plant tissues where it has broad, long-lasting toxicity to a variety of insect pests which feed on these plants [1, 2]. Neonicotinoids bind irreversibly, cumulatively [5] and with high affinity and specificity to the post-synaptic nicotinic acetylcholine receptors (nAChRs) within the central nervous system of insects, resulting in uncontrolled, excitatory depolarization of post-synaptic neurons [1, 6]. Due to the presence of a negatively charged nitro or cyano group, neonicotinoids have a much lower affinity for mammalian nAChRs and corresponding low toxicity to humans [1, 2]. Given their systemic nature, low doses of neonicotinoids are present in the nectar and pollen of crops grown from treated seed at mean maximum concentrations of 1.9 ng/g (~7.6 nM) in nectar and 6.1 ng/g (~24.4 nM) in pollen [7], which are sublethal for honey bees. On a typical foraging trip to a neonicotinoid-treated field, the quantity of neonicotinoids in pollen or nectar gathered by a worker honey bee would represent only 1–3% of its acute oral LD_50_ [7]. Cucurbit crops, exposed to neonicotinoids through a foliar spray or water treatment, may have higher neonicotinoid residues up to 122 ng/g (~488 nM) in pollen and 17.6 ng/g (~70 nM) in nectar [8]. Reported neonicotinoid residues in honey and pollen vary with geographic region; within our local area of Saskatoon, Saskatchewan, mean CLO and THI residues in honey were 8.2 ng/g (~33 nM) and 17.2 ng/g (~69 nM), respectively, based on 26 samples of honey and 19 samples of bee bread from 7 independent apiaries [4]. Saskatoon apiaries have 5–10 times higher neonicotinoid contamination of honey compared to the worldwide average of 1.8 ng/g (7.2 nM) neonicotinoids in honey [9]. Neonicotinoid contamination of honey and bee bread from apiaries in the Saskatoon area is common; greater than 50% of honey and bee bread contains CLO, while THI is detectable in 75% of honey and 21% of bee bread [4]. Within North America, CLO, THI, and IMD were detected concurrently in at least 50% of honey samples[9]. Considering the widespread neonicotinoid contamination of nectar, pollen, and agricultural wetlands [10], as well as the extended half-lives of neonicotinoids in soil [11], it is incumbent upon society to use this class of insecticides prudently to minimize pest resistance and non-target effects on pollinators and aquatic invertebrates [1, 12, 13].

The need for neonicotinoids to safeguard the crop health must be balanced with our reliance on pollinators for 35% of food production worldwide [14]. Managed European honey bee colonies (*Apis mellifera* Linnaeus), while contributing the greatest economic value in terms of pollination services [14], suffer from a variety of stressors, including *Varroa destructor* mites; viral, fungal, bacterial, microsporidial, and protozoan pathogens [15]; lack of genetic variability; declining and less diverse bee forage; climate change; and increases in trade and migratory beekeeping [16]. The role of sublethal neonicotinoid exposure in honey bee colony dysfunction is controversial. Laboratory experiments frequently report negative effects of field-realistic neonicotinoid concentrations on individual honey bees, ranging from impaired olfactory learning and long-term memory of honey bee foragers [17] to reduced sperm counts in honey bee queens [18]. In contrast, field studies often conclude that honey bee colonies foraging on neonicotinoid-treated crops have no difference in performance or vitality from colonies in untreated control fields [19]. A recent multi-country field study of honey bee colonies foraging on oilseed rape grown from neonicotinoid-treated seed found negative effects of CLO exposure on colony size and overwintering in some countries but not others [20]. Artificial feeding studies via sugar syrup and/or pollen patties are a method of controlled, colony-level exposure to neonicotinoids in the field. Outcome measures of these studies usually include colony-level inspection parameters such as brood area, adult bee population, colony weight, disease infestation, hygienic behaviour, pollen and honey stores, internal hive temperature and overwinter survival [21–27]. Results of artificial feeding studies have been mixed, with some authors observing high levels of overwinter mortality [23], others observing queen loss [24, 25, 27] and decreased social immunity [27]; and some studies finding no effect [21].

Despite conflicting scientific literature regarding neonicotinoids, some governments have gone ahead with policy decisions restricting their use. A ban on THI, CLO, and IMD has been in effect in the European Union since December 2013 for all agricultural crops which present a neonicotinoid-exposure risk for bees [28]. After the ban, European oilseed rape farmers experienced reductions in crop yield and quality, as well as increased production costs, leading to an estimated income loss of 880 million EUR per year for the oilseed rape industry [29]. Similarly, the provincial government in Ontario, Canada imposed new regulations to reduce the amount of corn and soybean seed treated with THI, CLO, or IMD by 80% in 2017 [30]. Citing safety concerns for aquatic invertebrates, the federal government of Canada is proposing to eliminate the use of IMD in agriculture over the next 3–5 years[31]. Considering the significant economic impact of neonicotinoid-use restrictions, there is a need for more reliable scientific evidence regarding the effects of neonicotinoids on honey bee colonies to justify these government policies.

The artificial feeding study presented here aims to address some of the deficiencies identified in the literature on sublethal neonicotinoid exposure of honey bees, including a lack of chronic, colony-level studies which utilize rapidly growing spring nucleus colonies and compare the effects of multiple neonicotinoids [32]. Chronic neonicotinoid toxicity studies are important to detect delayed sublethal effects on honey bee colonies because as length of neonicotinoid exposure time increases, the dose required to induce toxic effects decreases, in part due to the irreversible binding of neonicotinoids to nAChRs [33]. Surprisingly, there is a lack of protocols for chronic, sublethal neonicotinoid testing in honey bees to guide pesticide risk assessment by regulatory agencies and policy-makers [34, 35]. Existing regulatory guidelines for examining the chronic effects of pesticides on honey bee colonies specify a maximum 7 day pesticide exposure followed by a minimum colony observation period of 19–21 days [36, 37]. However, a recent Canadian study of honey bee colonies foraging near corn fields demonstrated that bees may experience continuous exposure to neonicotinoids through pollen for up to 4 months [27], suggesting that the current exposure times used in pesticide risk assessment may be insufficient. There is also a paucity of data on the sublethal effects of neonicotinoids on nucleus colonies, such as packaged bees, which are commonly used for hive replacement. These nucleus colonies may be more susceptible to the chronic, sublethal effects of neonicotinoids, as larger colonies are better able to detoxify and dilute pesticides within the hive [38].

The aim of our study was to compare the colony-level effects of chronic, sublethal exposure to the three most commonly used neonicotinoids (THI, CLO, IMD) on New Zealand packaged honey bees during spring and summer by measuring colony weight gain, capped brood area, and population size. Our study was able to demonstrate the effects of chronic, sublethal neonicotinoid exposure on the weight gain and cluster size of nucleus colonies, but not on brood area or adult population size.

## Materials and methods

### Ethics statement

Endangered or protected species were not used in this study.

### Experimental colonies

Sixty-eight 1 kg packages of honey bees, each with a queen of unknown genetic lineage, were obtained from Kintail Honey (Takapau, New Zealand) in association with Apiflora NZ Ltd (Tauranga, New Zealand) and were installed on April 25, 2016 at the Western College of Veterinary Medicine (WCVM) Goodale Research and Teaching Farm (52°01'50.6"N 106°32'26.6"W) within an approximately 0.2 km^2^ area within an alfalfa field surrounded by pasture and fields of canola and cereals. Permission was obtained from the WCVM Associate Dean’s Office for utilization of this study site. The colonies were installed in Langstroth standard (full depth) supers containing 10 Langstroth frames with plastic foundation and covered with a wooden lid with a central hole for feeding. The colonies were placed on leveled wooden pallets in groups of 2–4 with the pallets arranged several meters apart.

### Preparation

One and a half weeks prior to the start of the trial, the colonies were treated with oxytetracycline (Oxytet-25, Medivet Pharmaceuticals Ltd., High River, Alberta, Canada) and fumagillin (Fumagilin-B, Medivet Pharmaceuticals Ltd., High River, Alberta, Canada) in accordance with package instructions. All colonies were checked for the presence of a laying queen and the queen was marked. The colonies were provided with *ad libitum* 1:1 (w:w) sugar syrup and pollen patty (Ultra Bee Patties, Mann Lake Ltd., Hackensack, MN, USA) until the initiation of the trial. All colonies had 1.5–2 frames of brood at the start of the experiment and were thus considered uniform in colony strength. A plastic, front-mounted pollen trap (BeeMaid Honey, Winnipeg, MB, Canada) was placed on all colonies on May 13, 2016 (second week of the study) to coincide with the initiation of experimental pollen patty feeding.

### Experimental diet

100 mg/L stock solutions of thiamethoxam (99.6% purity; 37924, PESTANAL®, Sigma-Aldrich, Oakville, Ontario), imidacloprid (99.9% purity; 37894, PESTANAL®, Sigma-Aldrich, Oakville, Ontario), and clothianidin (99.9% purity; 33589, PESTANAL®, Sigma-Aldrich, Oakville, Ontario) were prepared in distilled water. Pollen patties were prepared from a mixture of 1 kg soybean flour and brewer’s yeast (3:2 ratio), 1.3–2 liters 2:1 (w:w) sucrose syrup and 0.5–2 kg (15.8–39.5% final patty weight) pollen obtained from pollen traps placed on colonies in 2015 and 2016. The quantity of pollen in the patties was increased over the course of the trial to satisfy the nutritional requirements of growing colonies. The neonicotinoid stock solutions were diluted in sucrose syrup (1:1 w:w) to a concentration of either 20 nmol/L (~5 ng/g) or 80 nmol/L (~20 ng/g). Neonicotinoid concentrations were calculated in molarity to obtain an equal number of molecules of each neonicotinoid in the experimental diet. Aliquots of the pollen patties and sugar solutions from treatment and control groups were submitted for measurement of neonicotinoid concentration by liquid chromatography-tandem quadrupole mass spectrometry (LC-MS/MS) (Government of Alberta Agriculture and Forestry Chemistry Laboratory, Edmonton, Alberta).

### Study design

The colonies were randomized into two treatment groups (20 nM, and 80 nM) for each of the three neonicotinoids (IMD, CLO, and THI) and a control group, with nine colonies per treatment group and fourteen control colonies. The results of a 2015 pilot study of colony weight gain in response to sublethal THI exposure were used to calculate the sample size for this study to achieve a power of 80% with a 95% confidence interval (OpenEpi, Version 3, open source calculator—SSMean).

Beginning May 7, 2016 until July 29, 2016 (12 weeks), 2.3 kg of experimental syrup was top fed to each colony per week using glass mason jars wrapped in tinfoil and covered by plastic pail (to protect from UV light) with three holes in each lid. Experimental pollen patty feeding began a week later, on May 13, 2016 until July 29, 2016. Pollen patties were changed weekly until the week of July 11, 2016 when biweekly patty feeding commenced to keep pace with colony consumption. The weight of pollen patties fed to the colonies was adjusted based on consumption of patties each week. Initially 300 g patties were provided, followed by a decrease to 180 g patties the week of May 20, a subsequent increase to 260 g patties the week of June 17, and a final increase to 500 g patties the week of July 1. The unconsumed syrup and patties were weighed at the end of each week to calculate total feed consumption per colony and total exposure to neonicotinoids expressed in micromoles.

The outcome measures for the trial included colony weight (as an estimate of honey production), brood area (as an estimate of reproduction), and number of adult bees and cluster size of adult bees (as an estimate of population size).

### Methods of measurement

The initial weights of the experimental colonies were measured on May 6, 2016 prior to exposure to neonicotinoids. The colonies were subsequently weighed at three-week intervals throughout the trial (weeks of May 23 [week 3], June 13 [week 6], July 4 [week 9], and July 25 [week 12]). The colonies were weighed in the early morning to maximize the number of bees within the hive, using a mechanical hanging scale (Salter Model 235, Brecknell Scales, Fairmont, MN, USA) with an accuracy to the nearest 0.5 kg. The final colony weights were corrected for any additional supers and frames used for colony expansion.

At week 12, digital images of both sides of all drawn frames in each colony were obtained using a 16.2 megapixel Nikon D7000 digital camera (Minato, Tokyo, Japan) mounted on a tripod with a Nikon 18–105 mm lens, a Nikon SU-800 wireless speedlight commander, Nikon SB-R200 wireless remote speedlights and a covered photo box made of white corrugated plastic (0.6 meters in length and slightly wider and higher than a single Langstroth frame). Each frame was placed in the photo box prior to image capture to standardize lighting conditions independently of the ambient lighting. The photos were taken in the morning to maximize the number of bees present inside each colony. The total area of capped brood for each frame was calculated from the photos based on the capped brood area detected by the HoneyBee Complete software (version 4.2, WSC Scientific GmbH, Heidelberg, Germany) using the auto-recognition function for capped brood and summing the result for all frames in each colony. Adult bees were not brushed from the frames prior to taking photos for brood recognition; however, the brood area was outlined manually for each frame, prior to auto-recognition of capped cells with the HoneyBee Complete software. For photo analysis of number of adult bees, the number of adult bees auto-recognized by the HoneyBee Complete software (version 5.4, WSC Scientific GmbH, Heidelberg, Germany) per frame was summed for each colony. Non-drawn frames were excluded from the analysis. For partially drawn frames, auto-recognition was limited to the areas of the frame with drawn wax. Percent recognition (number of auto-recognized bees/actual number of bees x 100) of the software calculated for four different types of frames (uncapped honey, capped honey, open brood, capped brood) was 63, 64, 82, and 69 percent respectively. The software underestimation was assumed to be the same across all colonies as photo conditions were consistent (same photo box and camera settings) for all colonies.

Cluster size as a measure of population size [39] was visually assessed at week 6 and week 12. Early in the morning, when bees were still clustered due to lower night temperatures in the prairies, a photo was taken of the top (week 6) or top and bottom (week 12) of each super. The number of interframe spaces (maximum of 11 for a 10 frame super) occupied by adult bees was determined visually to the nearest 0.25 for each super and summed for each colony at week 6. At week 12 the number of interframe spaces occupied on the top and bottom of each super was averaged and the averaged values summed for each colony. Repeatability of the cluster size measurements was assessed for a random sample of 25% of the colonies at week 12 and the Pearson Correlation Coefficient identified a high correlation between the repeated cluster size assessments (r (14) = 0.9947, p<0.001).

### Analysis of data

Five colonies were excluded from analysis due to queen failure including one colony from each of the 80 nM THI, 20 nM THI, and 20 nM CLO groups and two colonies from the control group, resulting in a sample size of 63. The twelve remaining control colonies were randomly assigned into the CLO, IMD, and THI groups for all factorial ANOVA analyses. All statistical analyses were performed using Stata/SE 14.2 or 15(College Station, TX). Normality was assessed using Shapiro-Wilk W test. Equality of variances was assessed using Bartlett’s test for equal variances or a two-sample variance comparison test. Data were presented as mean ± standard deviation (SD). Colony weight gain data were interpreted in two ways: (1) cumulative colony weight gain, defined as the difference between the colony weight at a given time point and the initial colony weight prior to the trial, and (2) three-week weight gain, defined as the difference between the colony weight at the indicated week and the colony weight three weeks prior. Similarly, consumption of syrup or pollen patty was analyzed as (1) cumulative consumption per colony over twelve weeks, and (2) weekly consumption per colony. Cumulative colony weight gain, capped brood area, number of adult bees, and cluster size data were analyzed with a 3x3 (dose x neonicotinoid) factorial analysis of variance (ANOVA) and a Bonferroni multiple comparison test with a p value <0.05 considered significant. Additional analysis of cumulative colony weight gain, as well as analysis of cumulative syrup and patty consumption were performed using a one-way ANOVA with a p value <0.05 considered significant. Specific post-hoc comparisons of the six neonicotinoid-dose treatment groups to the control were performed using a two-sample t-test with equal variances and a p value <0.01 considered significant, based on a Bonferroni correction for six comparisons [40].

Three-week colony weight gain and weekly syrup and patty consumption were analyzed with repeated measures mixed models with restricted maximum likelihood (reml) regression and a p value <0.05 considered significant. The model for three-week weight gain used an unstructured covariance matrix while the model for weekly consumption used an ar1 covariance matrix. The model for three-week weight gain was limited to weeks 6–12 and population size was included in the model by using the cluster size at week 6 as an estimate of the population at weeks 6 and 9 and the cluster size at week 12 as an estimate of the population at week 12. Specific post-hoc comparisons to the control group were performed using pairwise comparisons of predictive margins at chosen time intervals with a p value <0.01 considered significant.

Where no significant difference was observed, statistical power for comparing two means was calculated by the normal approximation method with a two-sided 95% confidence interval (OpenEpi, Version 3, open source calculator—PowerMean). A minimum of 80% statistical power to detect a difference was considered adequate.

## Results

### Colony weight gain

**Fig 1:**
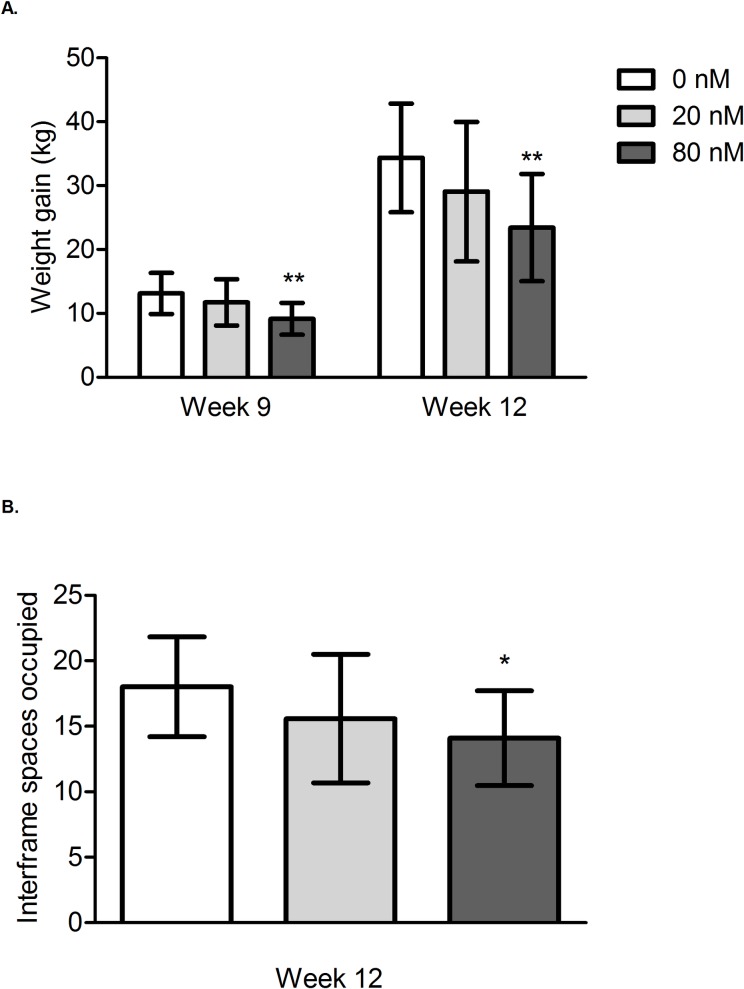
Chronic, sublethal neonicotinoid exposure decreases cumulative weight gain and cluster size of honey bee colonies. (A) Decrease in colony weight gain after exposure to 80 nM neonicotinoids for nine and twelve weeks and (B) decrease in colony cluster size after exposure to 80 nM neonicotinoids for twelve weeks. Treatment colonies were exposed to clothianidin (CLO), imidacloprid (IMD), or thiamethoxam (THI), at 20 or 80 nanomolar concentrations. The bars show mean cumulative colony weight gain (A) or cluster size (B) ± SD for each neonicotinoid dose group, which includes all three neonicotinoids tested. **significantly different from control, P<0.01. * significantly different from control, P<0.05.

**Fig 2:**
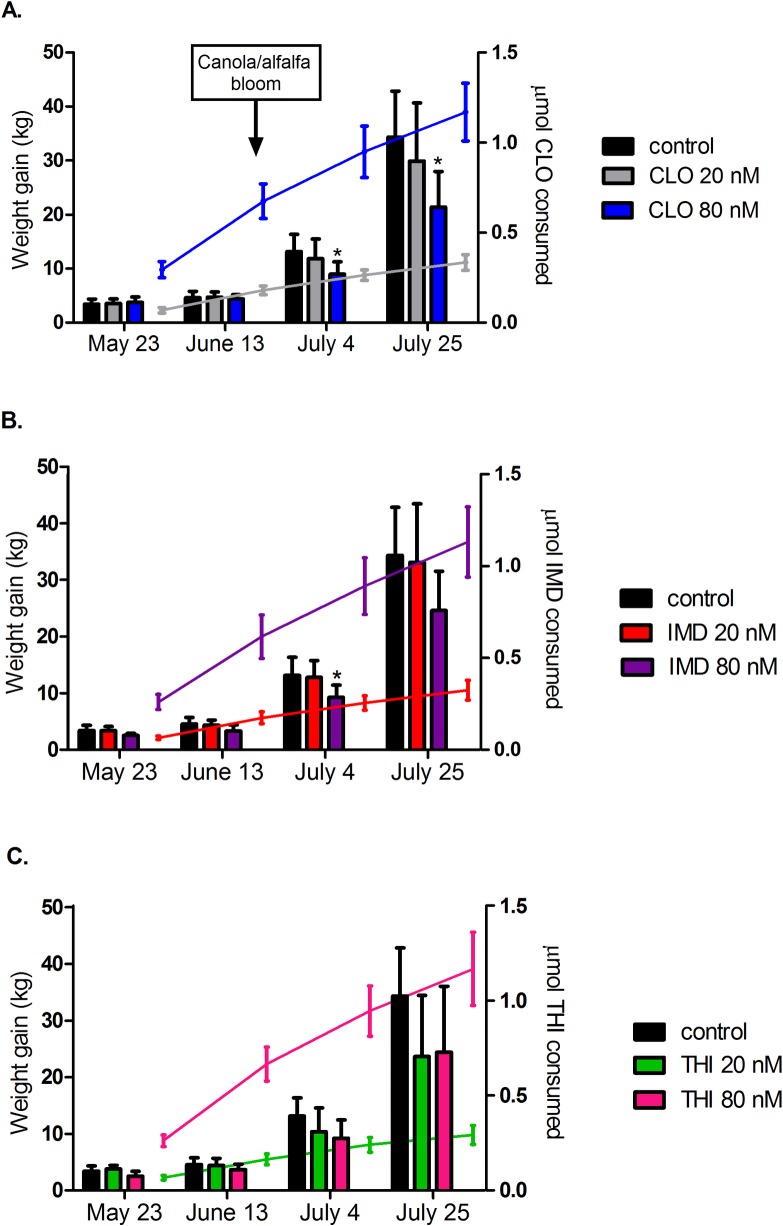
Cumulative weight gain of colonies exposed to sublethal doses of individual neonicotinoids for twelve weeks. Treatment colonies were exposed to clothianidin (CLO) (A), imidacloprid (IMD) (B), or thiamethoxam (THI) (C) at 20 or 80 nanomolar concentrations. Colonies exposed to 80 nM CLO (A) and 80 nM IMD (B) demonstrated significant decreases in weight gain from controls at weeks 9 and 12 and week 9, respectively. The bars show mean cumulative colony weight gain ± SD for each group (left y-axis). The curves show mean cumulative consumption of neonicotinoid per colony ± SD in micromoles for the treatment groups (right y-axis). * significantly different from control, P<0.01. The timing of the canola and alfalfa bloom surrounding the study site is indicated (A).

Colony weight gain data were analyzed by three different, although corroborating, methods including factorial and one-way ANOVA of cumulative weight gain data, and analysis of three-week weight gain data using a mixed model which accounted for differences in population size of the colonies. As described below, all analyses confirmed a significant negative effect of 80 nM doses of neonicotinoids on colony weight gain at weeks 9 and 12 with the 80 nM CLO group most frequently identified as the group with the lowest weight gain compared to the controls. Factorial ANOVA identified a significant effect of dose on cumulative weight gain at week 9 (*F*_2,54_ = 7.59, *p* = 0.0012) and week 12 (*F*_2,54_ = 5.79, *p* = 0.0053) with a nonsignificant interaction between dose and neonicotinoid treatment (*F*_4,54_ = 0.41, *p* = 0.8021 at week 9 and *F*_4,54_ = 0.74, *p* = 0.5711 at week 12), indicating that the effect of dose on weight gain was the same for all neonicotinoids tested. Colonies exposed to an 80 nM dose of IMD, THI, or CLO had a 30.2% and 31.8% decrease in cumulative weight gain compared to the controls at weeks 9 and 12 respectively (*p* = 0.002 at week 9 and *p* = 0.005 at week 12) (Fig 1A). Alternatively, analyzing cumulative weight gain data with a one-way ANOVA which compared individual neonicotinoid-dose treatments, significant differences in cumulative weight gain were identified at week 9 (*F*_6,56_ = 3.03, *p* = 0.0124) and week 12 (*F*_6,56_ = 2.82, *p* = 0.018). Compared to controls, cumulative colony weight gain was 31.9% and 37.8% lower after 9 and 12 weeks of exposure to 80 nM CLO, respectively, (Fig 2A [*t*_19_ = -3.3056, *p* = 0.0037 at week 9] [*t*_19_ = -3.7956, *p* = 0.0012 at week 12]) and 28.9% lower after 9 weeks of exposure to 80 nM IMD (Fig 2B [*t*_19_ = -3.0597, *p* = 0.0064]). Although statistically nonsignificant, colonies exposed to 80 nM IMD, 20 nM THI, and 80 nM THI for twelve weeks gained 28.3% (Fig 2B[*t*_19_ = -2.7982, p = 0.0115]), 31.2% (Fig 2C[t_18_ = -2.4830, p = 0.0231]), and 28.9% (Fig 2C[t_18_ = -2.2069, p = 0.0405]) less weight, respectively, compared to controls. At week 12, there was inadequate statistical power to detect a statistical difference in cumulative weight gain from control for the experimental groups treated with either 20 nM CLO, 20 nM IMD, 20 nM THI, or 80 nM THI. Mixed model analysis of three-week weight gain identified a significant interaction between neonicotinoid-dose treatment and time (χ^2^_12_ = 22.24, p = 0.0349), indicating that the effect of neonicotinoid-dose treatment on colony weight gain changed over time. Post-hoc analysis identified a significant difference of predicted three-week weight gain between the control and 80 nM CLO group from weeks 7–9 and weeks 10–12 (p = 0.002, p = 0.005).

### Population size and brood area

**Fig 3:**
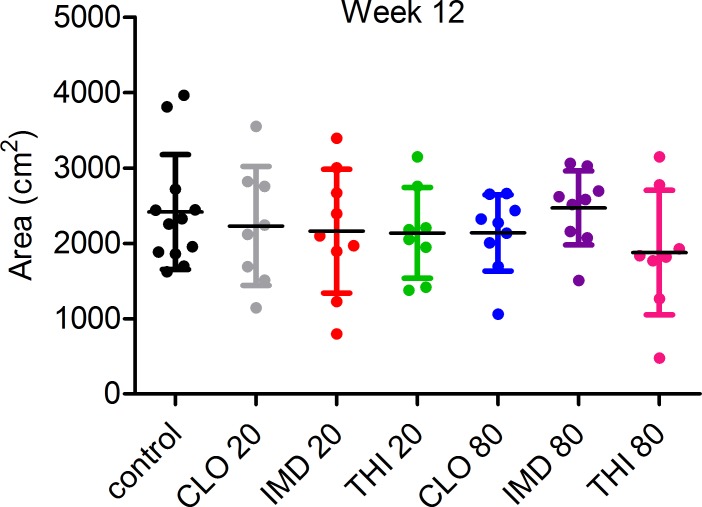
Capped brood area of colonies exposed to sublethal doses of neonicotinoid for twelve weeks. Treatment colonies were exposed for twelve weeks to clothianidin (CLO), imidacloprid (IMD), or thiamethoxam (THI) at 20 or 80 nanomolar concentrations. Brood area was quantified by analysis of digital images of brood frames with brood recognition software. There was no statistical difference among experimental groups but analyses lacked adequate (>80%) statistical power due to high variability. Mean ± SD are indicated for each group.

Twelve-week exposure to 80 nM neonicotinoids had a significant negative effect on cluster size of the colonies (Fig 1B), although these differences were not observed in the total adult bee counts or capped brood area. There was a significant effect of dose on cluster size at week 12 (*F*_2,54_ = 3.62, *p* = 0.0336) with a nonsignificant interaction between dose and neonicotinoid treatment (*F*_4,54_ = 0.31, *p* = 0.8705), indicating that the effect of dose on cluster size was the same for all neonicotinoids tested. Colonies exposed to an 80 nM dose had a 21.7% reduction in cluster size (p = 0.03) compared to the controls at week 12 (Fig 1B). There was no significant effect of dose (F_2,54_ = 1.78, p = 0.1781) or neonicotinoid treatment (F_2,54_ = 1.39, p = 0.2573) on the number of adult bees at week 12. Although statistically nonsignificant, colonies exposed to an 80 nM dose had 16.7% fewer adult bees compared to the controls at week 12 (S1 Database). Similarly, sublethal exposure to neonicotinoids did not have a significant effect on capped brood area after twelve weeks with no significant effect of dose (F_2,54_ = 0.61, p = 0.5497) or neonicotinoid treatment (F_2,54_ = 0.96, p = 0.3898) (Fig 3). However, analysis of both number of adult bees and capped brood area at week 12 lacked adequate (>80%) statistical power to detect an effect.

### Neonicotinoid consumption

**Fig 4:**
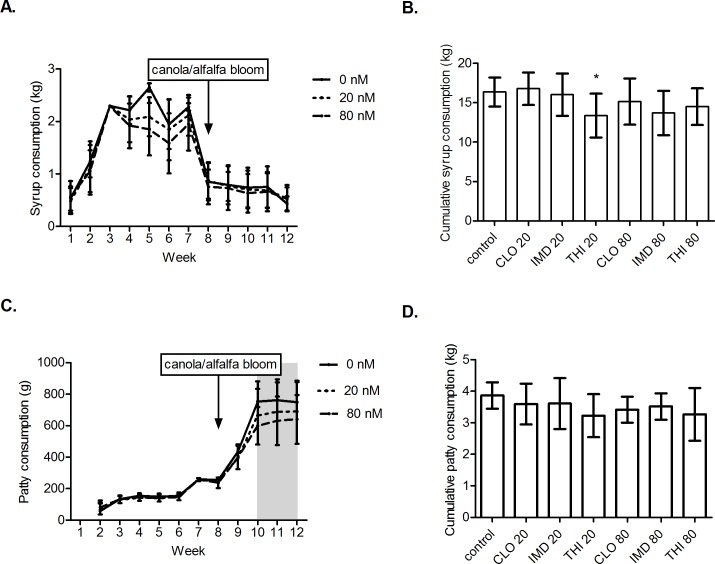
Weekly and cumulative feed consumption per colony over 12 weeks. Over twelve weeks, cumulative consumption of syrup (B) and pollen patty (D) was comparable for all experimental groups with the exception of colonies exposed to 20 nM thiamethoxam consuming 18.2% (2.98 kg) less syrup compared to controls. Shaded area indicates significant differences (P<0.01) in weekly pollen patty (C) consumption between control colonies and colonies exposed to 80 nM neonicotinoids. Treatment colonies were exposed to clothianidin (CLO), imidacloprid (IMD), or thiamethoxam (THI) at 20 or 80 nanomolar concentrations. Mean weekly (A, C) or cumulative (B, D) consumption per colony ± SD is indicated for each group. * significantly different from control, P<0.01. The timing of the canola and alfalfa bloom surrounding the study site is indicated (A, C).

The LC-MS/MS measured concentrations of neonicotinoids in experimental pollen patties and sugar syrup were on average 30% (SD 20.4%) below calculated concentrations in the experimental pollen patties and 0.075% (SD 18.6%) below calculated concentrations in the sugar syrup (S1 Table, S2 Table, S1 Database). A low level of THI contamination (~1 ng/g) was detected in one of each of the 80 nM CLO and 20 nM IMD pollen patty samples likely due to the addition of natural pollen in the experimental patties (S1 Table, S1 Database). Colony consumption of pollen patties and sugar syrup was analyzed by two complementary methods: (1) one-way ANOVA of cumulative consumption over the entire twelve week exposure period, and (2) mixed model analysis of weekly consumption over twelve weeks. Cumulative consumption of syrup (Fig 4B) and pollen patty (Fig 4D [F_6,56_ = 1.27, p = 0.2859]) was comparable for all experimental groups with the exception of colonies exposed to 20 nM THI consuming 18.2% (2.98 kg) less syrup compared to controls (F_6,56_ = 2.5, p = 0.0325; t_18_ = 2.9046, p = 0.0095). There was no significant difference in weekly consumption of sugar syrup among the 20 nM, 80 nM and control groups (Fig 4A [χ^2^_2_ = 4.81, p = 0.0901]); however, analysis of weekly patty consumption revealed a significant interaction between neonicotinoid dose (0, 20 or 80 nM) and week (Fig 4C [χ^2^_20_ = 61.74, p<0.001]), indicating that the effect of neonicotinoid dose on patty consumption was not constant over time. Colonies exposed to 80 nM neonicotinoids consumed 20.5% (154.2 g), 17.2% (130.9 g), and 14.5% (108.5 g) less patty than control colonies at weeks 10, 11, and 12 respectively (Fig 4C [p<0.001 at week 10, p<0.001 at week 11, and p = 0.002 at week 12]). There was a marked decline in syrup consumption at week eight coinciding with the bloom of canola and alfalfa in the surrounding environment and widespread availability of nectar (Fig 4A). At the same time, consumption of pollen patties increased rapidly in all colonies due to colony growth (Fig 4C). The installation of pollen traps on each colony promoted consumption of the experimental patties instead of pollen from the environment during colony expansion.

## Discussion

To our knowledge, this is the first colony-level study comparing sublethal effects of three neonicotinoids on honey bees. Similar to the colony-level artificial feeding study by Dively *et al*. [25] and Tsvetkov *et al*. [27] we chose a chronic, twelve-week exposure period, with sublethal neonicotinoid doses of 20 nM (~5 ng/g) and 80 nM (~20 ng/g), representing mid- and high-range, environmentally realistic concentrations respectively [25, 26]. The chosen neonicotinoid doses are similar to the calculated minimum and maximum residues of CLO [5.95 ng/g (~23.8 nM) and 19.04 ng/g (76.2 nM)] and THI [4.592 ng/g (~18.4 nM) and 19.29 ng/g (~77.2 nM)] in the pollen of oilseed rape grown from treated seed [41–43]. Our major finding was that chronic exposure of honey bee colonies to high environmental doses of neonicotinoids decreased colony weight gain by 30% compared to controls, which reflects predominantly honey production of the colonies (Figs 1 and 2). Similarly, Sandrock et al. [24] found that colonies chronically exposed to 2 ng/g (~8 nM) and 5 ng/g (~20 nM) CLO and THI in pollen patties collected significantly less pollen and produced 29% less honey; however, Faucon *et al*. [21] found no significant difference in weight gain of colonies fed syrup with 0, 0.5 ng/ml (~2 nM), and 5 ng/ml (~20 nM) IMD.

Decreased foraging activity, navigational ability, and longevity of worker honey bees [27, 44–46] of treatment colonies due to neurotoxic effects of neonicotinoids could explain the decreased honey production by the nucleus colonies exposed to 80 nM (equivalent to ~ 20 ppb or ng/g) neonicotinoids in our study. Correspondingly, Wu-Smart and Spivak [38] found that small colonies of 3000–7000 bees exposed to 10, 20, 50, and 100 ppb (~40, 80, 200, and 400 nM) IMD foraged less, although no difference in honey and nectar stores was observed, possibly due to the smaller size of the colonies, and the shorter experimental duration (3 weeks) compared to our study. At concentrations of 10 nM, IMD and CLO have been shown to inhibit the activity of Kenyon cells, neurons of the honey bee brain which are important in sensory processing for effective pollen and nectar collection [47]. Thus, the workers exposed to 80 nM neonicotinoids in our study may have had difficulty distinguishing and remembering floral odors, reducing their foraging success [17]. We cannot rule out that the presence of pollen traps may have weakened our experimental colonies; however, both treatment and control colonies experienced the same pollen restrictions to decrease potential dilution effects in the experimental diets. The 1 kg New Zealand packages of bees used in our study may have been more susceptible to negative effects of neonicotinoid exposure initially due to their small size. Had stronger colonies been used, a negative effect of neonicotinoid exposure on colony weight gain may not have been observed, as larger colonies may be better able to compensate for colony stress. Furthermore, unknown intake of neonicotinoids in nectar from surrounding fields is a limitation of this, and many other field studies. However, both treatment and control colonies had similar access to surrounding fields, and thus, treatment differences were attributed to the experimental diets, rather than neonicotinoid contamination from natural nectar sources. The use of natural pollen in our experimental pollen patties may also have been a source of neonicotinoid contamination (S1 Table); however, both treatment and control patties were prepared from the same source of natural pollen.

Nine to twelve weeks of sublethal exposure to neonicotinoids was required before a significant difference in colony weight gain was observed (Figs 1 and 2). This effect was only observed at the higher end of environmentally realistic dosages (80 nM) for IMD at week 9 (Fig 2B) and for CLO at weeks 9 and 12 (Fig 2A). At week 12, the colonies exposed to the mid- and high-range sublethal concentrations of THI experienced a statistically nonsignificant, approximately 30% lower cumulative weight gain (Fig 2C) compared the control group; however, there was inadequate statistical power to detect a difference from the control. Future studies with larger sample sizes or longer exposure duration would be desirable to confirm whether or not THI significantly impacts colony weight gain and honey production. Significant differences in colony weight gain among treatment and control colonies coincided with the bloom of alfalfa and canola in the surrounding environment. Rapid growth and increased foraging of the colonies in response to widespread availability of nectar may have allowed treatment differences to become more apparent. Thus, timing (for example, during nectar flow), rather than duration of exposure to neonicotinoids may be more important when designing colony-level exposure trials. Consumption of experimental syrup decreased during nectar flow (~week 8), resulting in decreased neonicotinoid exposure of treatment colonies despite an increase in pollen patty consumption (Fig 4). On average, colonies experienced a 25% decrease in total micromoles of neonicotinoid consumed per three-week interval from weeks 4–6 to weeks 7–9 (Fig 2).

Of the six neonicotinoid-dose combinations tested, colonies treated with 80 nM CLO experienced the greatest decrease in colony weight gain compared to controls, demonstrating 32% and 38% lower cumulative colony weight gain compared to controls after 9 and 12 weeks of exposure (Fig 2A). CLO has been shown experimentally to cause greater stimulation of the insect nAChR than IMD and cause larger neuronal depolarizations [47, 48], possibly explaining it’s more profound colony-level effects on weight gain. CLO also has the lowest acute 24-hour oral toxicity dose (3.35 ng/honey bee), followed by THI (4.4 ng/bee), and IMD (118.74 ng/bee) [49]. THI might be expected to have similar colony level effects as CLO, considering that THI is metabolized to CLO in insect and plant tissues [50]. In our study, the THI treated colonies, unlike the CLO treatment groups, did not demonstrate significant differences in colony weight gain compared to controls; however, our analysis lacked adequate statistical power to detect a difference. Although statistically nonsignificant, colonies exposed to 20 nM and 80 nM THI had 31% and 29% lower cumulative weight gain, respectively, compared to controls at week 12 (Fig 2C).

After twelve weeks of sublethal exposure to 80 nM of THI, CLO, or IMD, the adult bee cluster occupied 3.91 fewer interframe spaces in exposed colonies compared to controls (Fig 1B); however, unequivocal effects of neonicotinoid exposure on capped brood area were not demonstrated (Fig 3). Although the 80 nM-treated colonies exhibited decreases in both cluster size and adult bee numbers compared to controls; only the decrease in cluster size was statistically significant (p = 0.03). Inaccuracy of the software used for adult bee detection likely confounded analysis. Lack of statistical power further hindered characterization of population size and brood area in treatment groups compared to controls. Decreases in cluster size associated with exposure to 80 nM neonicotinoids could be explained by shortened lifespan of adult workers secondary to sublethal pesticide exposure during development in the brood comb [27, 46]. Decreased life expectancy and higher rates of forager loss as a result of compromised navigational ability [44, 45] could have a compounding negative effect on colony population size due to disruption of colony polyethism and resultant reduction in nurse bees available for brood care [24, 27, 46]. At peak consumption of the experimental diet, colonies exposed to 80 nM neonicotinoids consumed significantly less pollen patty compared to control colonies (131.2 g less pollen patty on average from weeks 10–12 [Fig 4C]). This could be partially explained by the decreased population size (as estimated by cluster size) of the 80 nM exposed colonies. Decreased consumption of the experimental patties by treatment colonies is unlikely to be the result of an ‘antifeedant effect’ of sublethal neonicotinoid doses based on published field and laboratory studies [21, 26, 51]. Similarly, we could not demonstrate differences in consumption of colonies simultaneously offered four doses (0, 4, 40, and 400 nM) of one of three neonicotinoids (IMD, CLO, or THI) in syrup (unpublished data). Differences in mean cumulative syrup consumption per colony between treatment group and control groups (maximum difference of 2.99 kg between controls and colonies exposed to 20 nM THI [Fig 4B]) were not large enough to explain the differences in cumulative weight gain observed in the treatment colonies compared to controls (Figs 1 and 2).

The significant decrease in cluster size after 12 weeks of 80 nM neonicotinoid exposure in our study contrasts with the absence of an observable effect of sublethal neonicotinoid exposure on brood area, and further contributes to the often conflicting results of previously published artificial feeding studies of IMD to honey bee colonies. Dively *et al*. [25] exposed colonies to 5, 20, and 100 ng/g (~20, 80, and 400 nM) IMD in pollen patties for 12 weeks and found no difference in capped brood or population size associated with treatment. Similarly, Meikle *et al*. [26] demonstrated no difference in capped brood area among colonies exposed to 5 and 20 ppb (~20 and 80 nM) IMD for 6 weeks in sugar syrup, although colonies exposed to the environmentally unrealistic dose of 100 ppb (~400 nM) IMD had a significant decrease in brood area. Faucon *et al*. [21] exposed strong colonies to 0.5 ng/ml and 5 ng/ml (~2 and 20 nM) IMD in sugar syrup for 34 days and found no difference in cluster size or capped brood during the experiment. In contrast, Wu-Smart and Spivak [38] found that exposure of small colonies (<10 000 bees) to 20, 50 or 100 ppb (~80, 200, and 400 nM) of IMD in sugar syrup for three weeks had a negative impact on brood quantity and pattern. Sandrock *et al*. [24] found that strong colonies exposed to a combination of 5 ppb (~20 nM) THI and 2 ppb (~8 nM) CLO in pollen patties for 46 days had 13% less brood and 28% fewer adult bees compared to controls at the end of the exposure period. The social organization of honey bee colonies allows them to be resilient to stress [25]. The addition of multiple, concurrent stressors, such as cold temperature or disease, may exacerbate sublethal effects of neonicotinoids on brood area or population size, explaining the often incongruent findings reported in the literature [7, 20]. There may have been a qualitative effect on brood area in the neonicotinoid-exposed colonies in our study; however, this sublethal reproductive effect may have been obscured by increased investment in brood production by exposed colonies [7, 32, 52]. Furthermore, high variation in the brood area data led to a lack of statistical power and higher than accepted probability of type II error. Prior to the start of our study, both treatment and control colonies received oxytetracycline treatment in accordance with recommended beekeeping practice for installation of New Zealand packaged bees in Canada. Oxytetracyline has been shown to cause significant elevations in brood mortality [53] as well as decreased diversity of the honey bee gut microbiome leading to increased susceptibility to opportunistic pathogens [54]. In our study, any negative effects of oxytetracycline treatment on colony weight gain, brood area or population size and health should have been experienced equally by control and treatment colonies. Considering that the majority of honey bee colonies in North America receive antibiotic metaphylaxis for American foulbrood, the oxytetracycline treatment of our study colonies is representative of the iatrogenic stress experienced by most North American honey bees. It is important to note that administration of antibiotics to honey bees is not permitted in some jurisdictions outside of North America (e.g. European Union).

One of the major observations in our study was the large amount of variation in our experimental colonies which undermined the statistical power of the colony weight gain, brood area and population size analyses. New Zealand packages were chosen as experimental colonies to standardize colony strength at the beginning of the study; however, the colonies did not have sister queens, introducing genetic variation among colonies. Sandrock *et al*. [24] found that colonies from different genetic lineages of *A*. *mellifera* differed in their susceptibility to chronic exposure of THI and CLO. Variability in colony genetics could influence the ability of individual bees to detoxify neonicotinoids [21]. Our initial sample size of nine colonies per treatment group was chosen based on results of a previous pilot study of THI exposure on colony weight gain and was similar to the sample size of other artificial feeding studies [21, 25]; however, some authors have recommended three to nine times greater samples sizes to compensate for the inherent variability of honey bee colonies [55–57]. Lack of statistical power is not a problem unique to our study and is present in many other studies of sublethal effects of neonicotinoids [32, 56], emphasizing the need for more sensitive and specific tests [56, 57].

## Conclusion

Similar to other studies [25], we found largely no effect on colony performance at the mid-range doses (20 nM or ~5 ng/g) of neonicotinoids present in the environment. Negative effects of sublethal exposure to neonicotinoids on honey production and cluster size were observed only after 9–12 weeks of exposure to the higher-end of environmentally realistic concentrations (80 nM or ~20 ng/g). Although concentrations of 80 nM neonicotinoids have been documented in honey samples from our local area [4], this concentration is 10 times higher than the average maximum neonicotinoid concentrations in nectar based on a review by Godfray *et al*. (2014) [7]. Production of a commodity, in this case honey, is a common method to assess toxicity in food producing species, such as monitoring milk production to understand ergot toxicity in dairy cattle [58]. Honey production is also an economically relevant parameter for beekeepers and for farmers who rely on the foraging activity of honey bees for crop pollination [32]. The significant differences in weight gain observed in the colonies exposed to 80 nM neonicotinoids in our study suggest that honey production is a useful colony-level parameter to estimate sublethal neonicotinoid exposure in honey bees.

## Supporting information

S1 Table Neonicotinoid concentrations within experimental pollen patties. (DOCX)

S2 Table Neonicotinoid concentrations within experimental syrup. (DOCX)

S1 Database Raw data for all figures and analyses including colony weight gain, cluster size, capped brood area, number of adult bees, syrup consumption, patty consumption, neonicotinoid consumption, and measured neonicotinoid concentrations in syrup and patties. (XLSX)
