# Supplementary material for: Comparative chronic toxicity of three neonicotinoids on New Zealand packaged honey bees
Source: PLoS One. 2018 Jan 2;13(1):e0190517. doi: 10.1371/journal.pone.0190517 (PMC5749814; doi:10.1371/journal.pone.0190517)
Supplement: S1 Table — (DOCX) [file pone.0190517.s001.docx]

**S1 Table. Neonicotinoid concentrations within experimental pollen patties.**

| **Experimental diet (nM)** | **Sample size (n)** | **Calculated concentration (ng/g)** | **Mean LC-MS/MS measured concentration ± SD (ng/g)** | **Mean percent difference from expected ± SD** **(%)** | **THI contamination (ng/g)** |
| --- | --- | --- | --- | --- | --- |
| control | 3 | 0 | 0 | 0 ± 0 |  |
| CLO 20 | 4 | 5 | 3.7 ± 0.36 | -26 ± 7.12 |  |
| CLO 80 | 4 | 20 | 13.9 ± 0.70 | -30.5 ± 3.49 | 1.2, n=1 |
| IMD 20 | 4 | 5 | 2.6 ± 0.95 | -47.5 ± 19.07 | 1.1, n=1 |
| IMD 80 | 4 | 20 | 9.9 ± 3.32 | -50.5 ± 16.60 |  |
| THI 20 | 3 | 5 | 4.6 ± 0.47 | -7.33 ± 9.45 |  |
| THI 80 | 3 | 20 | 18.1 ± 3.75 | -9.5 ± 18.76 |  |

CLO, clothianidin; IMD, imidacloprid; THI, thiamethoxam; LC-MS/MS, liquid chromatography-tandem quadrupole mass spectrometry. Percent difference from expected was calculated using: $\frac{(\left[ measured \right]-\left[ calculated \right])}{[calculated]}\times100\%$.
