# Supplementary material for: Comparative chronic toxicity of three neonicotinoids on New Zealand packaged honey bees
Source: PLoS One. 2018 Jan 2;13(1):e0190517. doi: 10.1371/journal.pone.0190517 (PMC5749814; doi:10.1371/journal.pone.0190517)
Supplement: S2 Table — (DOCX) [file pone.0190517.s002.docx]

**Table S2.** **Neonicotinoid concentrations within experimental syrup.**

| **Experimental diet (nM)** | **Sample size (n)** | **Calculated concentration (ng/g)** | **Mean LC-MS/MS measured concentration ± SD (ng/g)** | **Mean percent difference from expected ± SD (%)** |
| --- | --- | --- | --- | --- |
| control | 3 | 0 | 0 | 0 ± 0 |
| CLO 20 | 3 | 5 | 4.4 ± 0.56 | -12 ± 11.14 |
| CLO 80 | 3 | 20 | 18.6 ± 0.95 | -6.83 ± 4.75 |
| IMD 20 | 4 | 5 | 5.5 ± 1.36 | 9 ± 27.25 |
| IMD 80 | 3 | 20 | 18.7 ± 1.88 | -6.67 ± 9.39 |
| THI 20 | 3 | 5 | 5 ± 0.53 | -5.92 x 10^-15^ ± 10.58 |
| THI 80 | 4 | 20 | 22 ± 5.50 | 9.75 ± 27.49 |

CLO, clothianidin; IMD, imidacloprid; THI, thiamethoxam; LC-MS/MS, liquid chromatography-tandem quadrupole mass spectrometry. Percent difference from expected was calculated using: $\frac{(\left[ measured \right]-\left[ calculated \right])}{[calculated]}\times100\%$.
